# Age Related Osteoporosis: Targeting Cellular Senescence

**DOI:** 10.3390/ijms23052701

**Published:** 2022-02-28

**Authors:** Ursula Föger-Samwald, Katharina Kerschan-Schindl, Maria Butylina, Peter Pietschmann

**Affiliations:** 1 Medical Science and Human Medicine Study Programme, Karl Landsteiner University of Health Sciences, 3500 Krems an der Donau, Austria; 2 Department of Physical Medicine and Rehabilitation, Medical University of Vienna, 1090 Vienna, Austria; katharina.kerschan-schindl@meduniwien.ac.at; 3 Department of Pathophysiology and Allergy Research, Medical University of Vienna, 1090 Vienna, Austria; maria.butylina@meduniwien.ac.at (M.B.); peter.pietschmann@meduniwien.ac.at (P.P.)

**Keywords:** cellular senescence, age-related osteoporosis, osteoporosis therapy

## Abstract

Age-related chronic diseases are an enormous burden to modern societies worldwide. Among these, osteoporosis, a condition that predisposes individuals to an increased risk of fractures, substantially contributes to increased mortality and health-care costs in elderly. It is now well accepted that advanced chronical age is one of the main risk factors for chronical diseases. Hence, targeting fundamental aging mechanisms such as senescence has become a promising option in the treatment of these diseases. Moreover, for osteoporosis, the main pathophysiological concepts arise from menopause causing estrogen deficiency, and from aging. Here, we focus on recent advances in the understanding of senescence-related mechanisms contributing to age-related bone loss. Furthermore, treatment options for senile osteoporosis targeting senescent cells are reviewed.

## 1. Introduction

It is common clinical knowledge that geriatric patients typically suffer from several diseases such as atherosclerosis, diabetes mellitus, cataracts, cognitive impairment, sarcopenia, osteoarthritis, and osteoporosis. Osteoporosis is the most frequent type of metabolic bone diseases and may lead to fragility fractures and associated morbidity and mortality [1]. Although the above-mentioned age-related diseases affect distinct organ systems and lead to very different disease manifestations, over time comprehensive pathophysiologic concepts emerged. As examples of such concepts, inflammaging and the acquisition of a “senescence-associated phenotype” (SASP) by aging cells should be mentioned [2,3,4]. In this narrative review, we introduce the reader to the biology and pathophysiology of bone with a special focus on cellular senescence. In the final part of our manuscript, we discuss how age-related osteoporosis could be treated by targeting the SASP.

## 2. Bone Biology

The human skeleton is defined as an adaptive structure, which undergoes remodeling throughout the whole life. This process is important for maintaining bone strength, as old and micro damaged bone is exchanged with mechanically stronger one. In general, trabecular and cortical bone can be distinguished. Trabecular bone is composed of a honeycomblike network, comprising trabecular plates and rods. In contrast, cortical bone is solid, dense, and surrounds the marrow space. Both types of bone comprise four types of different cells: osteoblasts, osteocytes, bone lining cells, and osteoclasts [5,6,7].

### 2.1. Bone Cells

**Figure 1:**
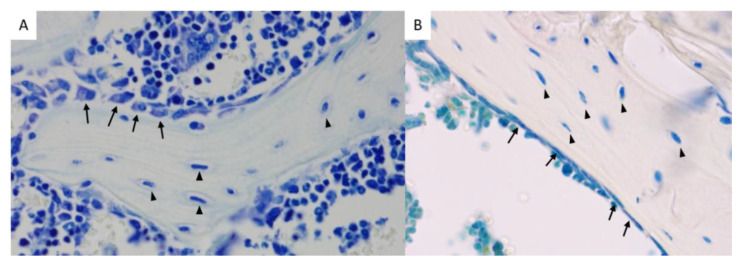
(**A**) Toluidine blue staining of a mouse femur showing osteoblasts (arrows) and osteocytes (triangles) (female, 8 weeks of age, original magnification 400×). (**B**) TRAP and toluidine blue staining of a mouse tibia, showing bone lining cells (arrows) and osteocytes (triangles) (female, 18 weeks of age, original magnification 400×).

Mature osteoblasts are located along the bone surface and are responsible for bone formation by secretion of bone matrix proteins and guidance of mineralization (Figure 1). These cuboidal cells originate from pluripotent mesenchymal stem cells (MSCs) and comprise 4–6% of the total resident bone cells. Two major signalling pathways, activated by wingless-related integration site (Wnt) proteins and bone morphogenic proteins (BMPs), are responsible for osteoblast differentiation. In contrast, Dickkopf-1, sclerostin, IL-6, and TNFα are known to be negative regulators of bone forming cells After finishing bone formation, a subset of mature osteoblasts differentiates into osteocytes, which are encapsulated within the newly formed bone matrix [5,7,8,9]. Osteocytes lie in so called lacunae throughout mineralized bones, where they are responsible for supporting bone structure and metabolism (Figure 1). Mechanosensation is described to be their main function, as osteocytes transduce stress signals from bending or stretching bone into biologic activity. They are considered as the most abundant type of cells (90%) found within the matrix or on bone surfaces. Furthermore, osteocytes express macrophage colony-stimulating factor (M-CSF) and receptor activator of nuclear factor-kB ligand (RANKL), which stimulate preosteoclast proliferation and induce osteoclast differentiation, and they express sclerostin, an inhibitor of osteoblastogenesis [5,7]. Osteoblasts, which have completed bone matrix-synthesis, but have not been encapsulated, undergo apoptosis or become inactive bone-lining cells (Figure 1) [5,7,8,9]. These flat shaped cells cover the quiescent surface of the bone, where neither bone resorption nor bone formation occurs. They are involved in osteoclast differentiation as they produce osteoprotegerin (OPG) and RANKL. Furthermore, bone lining cells are able to block the immediate interaction between osteoclasts and the bone matrix, when bone should not be degraded [5,7,8].

**Figure 2:**
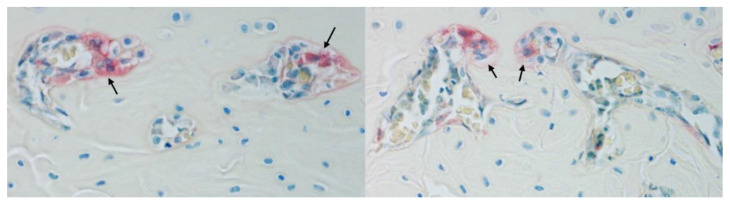
TRAP and toluidine blue staining of a Sprague-Dawley rat tibia, showing osteoclasts with positive TRAP staining (arrows) (female, 10–12 weeks of age, original magnification 400×).

Multinucleated osteoclasts are the primary cells involved in bone resorption (Figure 2). M-CSF and RANKL are considered as two important cytokines involved in osteoclast formation, differentiation, and survival. Furthermore, IL-1, IL-6, IL-17, and TNF-α enhance osteoclast generation and bone resorption. In contrast, RANKL decoy receptor OPG, IFN-γ, IL-3, IL-4, IL-10, and IL-12 are known as negative regulators of bone resorbing cells. A dysregulated activity of osteoclasts leads either to an increased or reduced bone mass [7,8].

### 2.2. Bone Modelling and Remodelling

Bone modelling is responsible for bone development and bone growth and is performed by osteoblasts and osteoclasts, which function independently. Osteoblasts are responsible for bone formation and osteoclasts for resorption, which leads to a shape alteration of existing bone. The process of modelling occurs predominantly during skeletal growth before achievement of peak bone mass [7,10].

Additionally, bone is remodeled throughout the entire life. Bone remodeling is characterized as a highly complex process during which old bones are renewed for maintaining bone strength and mineral homeostasis. Responsible for bone remodeling is the tightly linked action of osteoblasts and osteoclasts. The remodeling cycle is initiated by the degradation of old bone by multinucleated osteoclasts, a process that requires about 2–4 weeks. In the following reversal phase osteoclasts undergo apoptosis, whereas osteoblasts synthesize the organic extracellular bone matrix and coordinate its mineralization. The whole bone formation process takes about four to six months to achieve. Fracture healing, preservation of bone mechanical strength by replacing older and micro-damaged bones with healthier ones, skeleton adaptation to mechanical use, and calcium and phosphate homeostasis are described as the main functions of bone remodeling. Underlining its importance, imbalances of bone resorption and formation during the process of bone remodeling may lead to several bone diseases, such as, for example, osteoporosis [5,7,11,12].

### 2.3. Age-Related Osteoporosis

Osteoporosis is defined as “a frequent age-related disease, which is associated with a low bone mineral density (BMD) and a systemic impairment of bone mass and microarchitecture that predisposes a person to an increased risk of fracture” [13]. It initially proceeds asymptomatic, until fractures of the hip, wrist, and vertebrae occur, which are considered as the typical clinical manifestation of osteoporosis. They are not only painful and cause deformity and disability, but also increase mortality [1,9,11,14]. According to the World Health Organization (WHO), osteoporosis is defined as a BMD that is 2.5 standard deviations (SD) below the mean for young healthy adults of the same sex (T-score < −2.5)”. T-scores in the range of −1 and −2.5 indicate osteopenia and affected persons show an increased risk of developing osteoporosis [1,15].

Generally, osteoporosis can be divided into primary and secondary osteoporosis. The first one can be further divided into postmenopausal and senile or age-related osteoporosis. Osteoporosis occurs in all age groups, gender, and races, but is more frequently found in women and older men. Whereas postmenopausal osteoporosis arises due to estrogen deficiency, senile osteoporosis is associated with aging processes including inflammatory processes, increased parathyroid hormone levels, calcium and vitamin D insufficiency, or osteoblast dysfunction. Secondary osteoporosis is related with bone loss due to a secondary underlying condition or intake of specific medication [1,15,16,17]. 

The diagnosis of osteoporosis comprises several steps, including medical history, physical examination, undergoing a bone density test, and the performance of blood and urine tests. Assessment of bone mineral density is usually conducted by dual-energy absorptiometry (DXA), which is considered as gold standard in osteoporosis diagnosis. Another diagnostic tool is the so-called Fracture Risk Assessment Tool (FRAX), which is available online. It identifies appropriate patients for treatment by considering risk factors such as age, race, gender, body mass index, prior personal, or parental history of fractures, and many others. In clinical practice, FRAX is used in addition to other diagnostic tools such as DXA, but is known to have some limitations [1,16,18]. 

## 3. Cellular Senescence

Cellular senescence describes a state of irreversible or stable cell arrest maintained even in the presence of mitogenic stimuli. It was first discovered by Hayflick and Moorhead 70 years ago, when they described that in vitro serially cultured primary human fibroblasts stop dividing after a certain number of passages [19,20]. This observation of a limited potential to replicate is now well known as “Hayflick limit” or replicative senescence. Since then, the concept of cellular senescence has been gradually expanded and now encompasses a highly heterogeneous phenomenon of irreversible or stable cell arrest driven by multiple mechanisms. Moreover, it is now well established that senescent cells play key roles in physiological as well as pathological processes with both beneficial and detrimental effects (for review see [3,4]).

A common feature of senescent and quiescent cells is the loss of proliferative capacity. However, what distinguishes these states of cell cycle arrest is that quiescent cells can re-enter the cell cycle in response to appropriate mitogenic signals, whereas in senescent cells, the non-proliferative state in most cases is an irreversible one. An exception to this is seen in senescent tumor cells, which, given certain circumstances, are capable of resuming proliferative activity [21,22,23,24,25]. Another key feature distinguishing senescent form quiescent cells is halted proliferation combined with still ongoing cell growth. Senescent cells are characterized by an increased cell size but also increased metabolic and lysosomal activity. Moreover, senescent cells acquire a “senescence-associated secretory phenotype” (SASP). They release a mixture of secreted factors, e.g., proinflammatory cytokines, chemokines, growth modulators, angiogenic factors, or matrix metalloproteases. Thereby, they interact with and influence surrounding cells in a situation-dependent manner and initiate various effects including tissue repair, tissue regeneration, promotion of stemness and tissue plasticity, or clearance by cells of the immune system (for review see [3,4]). 

The factors released by senescent cells can also reinforce senescence in an autocrine fashion or activate senescence in neighboring cells in a paracrine fashion, a state referred to as paracrine senescence [26]. Thus, senescent cells, in contrast to cells undergoing apoptosis, remain active for a long period of time before being eliminated. They are transformed into cells with increased metabolic activity capable of changing their microenvironment and, thereby, actively influencing physiological processes. Recent data suggest that the senescent cell secretome impacts not only its microenvironment but also regions more distant. In addition to being released into the direct surrounding of cells, SASP-components have been shown to be part of small exosome-like extracellular vesicles (EVs) [27]. Thereby, information can be passed in a cell-to-cell or organ-to-organ manner in a process termed EV trafficking. A role of EVs in cell-to-cell communication has been shown in various models [28,29]. A role of EVs in distant communication between organs has only recently been demonstrated by Jiang et al. [30]. They provided evidence for hepatocyte-derived miR-1 packed into and transferred via EVs to promote endothelial inflammation and facilitate atherogenesis.

### 3.1. Different Types of Cellular Senescence

Depending on the triggering stimulus, different types of senescence are usually defined. Progressive telomere shortening associated with DNA end-replication is the underlying mechanism of the type of senescence observed by Hayflick and Moorhead [19,20] and is now referred to as replicative senescence. Shortening of chromosomes as a result of recurring cell divisions activates the DNA damage response (DDR) and, finally, leads to cell cycle arrest. DNA damage triggering a senescent state in cells can, besides changes in telomers, have many reasons and non-telomeric DNA damage-induced senescence is referred to as stress-induced cellular senescence. It includes, for example, oncogene-induced senescence (OIS), therapy-induced senescence (TIS) in tumor cells, senescence induced by epigenetic changes, or senescence linked to mitochondrial dysfunction. Finally, the presence of senescent cells in developing organs suggests a role in organic and embryonic development, and the type of senescence seen in this context is referred to as programmed cell senescence (for review see [3,4]). Irrespective of the triggering stimulus, a central molecular mechanism of cellular senescence is the increased expression of the cyclin dependent kinase (CDK) inhibitors p21^Wafl^ and p16^INK4a^, which in turn activates the retinoblastoma (RB) family of proteins and initiates cell cycle arrest (for review see [3]). The SASP, playing a key role in mediating beneficial as well as detrimental effects of senescent cells, varies considerably in composition and strength, depending, e.g., on the initial trigger, the type of cell undergoing senescence, or on whether the cell is in an early or late stage of senescence. Likewise, mechanisms leading to and regulating the SASP are also highly heterogeneous and complex. Two transcription factors, NF-κB and CEBPβ, have emerged as key players and regulators of SASP protein expression, with proinflammatory cytokines IL-6 and IL-8 being the most robustly expressed (for review see [3]). However, an in-depth understanding of the highly dynamic process of SASP protein expression is essential for successful manipulation of senescence-associated processes for therapeutic purposes. A valuable contribution to a comprehensive characterization of context specific SASP components was only recently provided by Basisty et al. with a “SASP Atlas”, a first proteome-based database of soluble proteins and exosomal cargo proteins that are part of the SASP [31].

### 3.2. Aging and Senescence

Several studies provide evidence for an accumulation of senescent cells in tissues of aging organisms [32], suggesting a role of senescence in tissue dysfunction and age-related pathologies such as, e.g., cancer, osteoarthritis, osteoporosis, or Alzheimer’s disease. Another hallmark of aging is a chronic low-grade inflammation even in the absence of acute infection [2]. The predominantly proinflammatory nature of the SASP is thought to link senescence with this state of “inflammaging” by persistently releasing proinflammatory cytokines. An additional link between senescence and the immune system proposes a per se protective role of senescence and a growing inefficiency of the immune system in aging organisms leading to a failure in removing senescent cells from tissues. A persistent presence of senescent cells finally turns initially favorable into unfavorable effects and leads to pathologies. Taken together, an aging immune system fails to clear tissues from senescent cells, which, in turn, promotes a state of chronic inflammation [4,33,34]. The direct involvement of senescent cells in chronic low-grade inflammation and age-related pathologies has been demonstrated in several murine models [35,36,37,38,39,40,41,42]. Jeon et al. for instance, provided evidence for attenuation of post-traumatic osteoarthritis by clearance of senescent cells in p16-3MR transgenic mice [37]. In a very recent study of Childs et al., clearance of senescent cells in the *Ldlr*^−/−^ mouse model of atherosclerosis reinforced fully deteriorated fibrous caps that normally prevent atherogenic plaque rupture [41]. Collectively, these findings support the usefulness of senescent cells as therapeutic targets for the treatment of age-related pathologies.

### 3.3. Age-Related Osteoporosis and Senescence

As in other age-related diseases, research in the last decade has clearly provided evidence for a role of senescence in age-related osteoporosis (for review see [43,44,45,46,47,48]). In pioneering work by Farr et al. [49] the expression of the senescent cell biomarker p16^Ink4^ was shown to increase in bone-derived B cells, T cells, myeloid cells, osteoprogenitors, osteoblasts, and osteocytes from young versus old male and female mice. Moreover, osteocytes and myeloid cells were identified as the cell populations with the most pronounced upregulation of SASP factors within the bone environment [49]. Accumulation of senescent cells in the context of age-related and radiotherapy-related bone loss was since then confirmed by others [50,51], and was also shown in bone biopsy samples from older postmenopausal compared to younger premenopausal women [49]. A causative role of senescent cells in mediating age-related bone loss was finally evidenced by pharmacological clearance of senescent cells in old mice or genetic clearance of senescent cells by inducible elimination of p16^Ink-4a^-expressing senescent cells using INK-ATTAC transgenic mice [42]. The positive effect on bone microarchitecture and bone strength observed in these models after clearance of senescent cells was shown to be mediated partly by the elimination of senescent osteocytes. Moreover, increased bone formation by osteoblasts and a reduction in bone marrow adipose tissue was seen, and thereby supported a shift in bone marrow-derived mesenchymal stem cell (BMSC) differentiation from osteoblasts to adipocytes as mechanism of senescence mediated age-related bone loss [42]. 

Mechanisms underlying senescent induced changes in MSCs have since then been addressed in multiple studies, identifying important mediators of MSC senescence [52,53,54,55,56,57,58,59,60,61,62,63]. For instance, several miRNAs have been identified that are involved in regulating senescence and the balance between osteoblastic and adipocytic differentiation of BMSCs [52,55,60,61]. Lian et al. investigated an involvement of miR-29a in age-related bone loss [61]. Knockout and overexpression of miR-29a was associated with exacerbated and reduced bone loss, respectively, in old mice. Oxidation resistance protein-1 (*Oxr1*) and forkehead box O3 (*FoxO3*) were identified as target genes, suggesting an antioxidative effect of miR-29a associated with reduced osteoblast senescence [61]. A miRNA thought to be involved in regulating the shift between osteogenic and adipogenic differentiation is miR-499 [60]. Wu et al. revealed a central role of the long coding RNA zing finger antisense 1 (ZFAS1) in this process by showing that *ZFAS1* knockdown in BMSCs facilitated osteogenic differentiation and at the same time suppressed cell senescence. Moreover, they identified downregulation of miR-499 and consequently upregulation of ephrin type-A receptor 5 (EPHA5) as downstream events targeted by ZFAS1 and promoting the adipogenic potential of BMSCs. In vivo, *ZFAS1* knockdown was associated with increased bone mass [60]. Finally, two groups investigated mechanisms involved in regulating osteogenesis and adipogenesis in BMSCs in vitro and identified miR-363-3p [54] and miR-245a [55] as regulators of senescence. miR-363-3p targets tumor necrosis factor receptor-associated factor 3 (TRAF3), which promotes BMSCs differentiation to osteoblast and suppresses senescence and differentiation to adipocytes [54]. miR-145a was shown to be upregulated by p53, a positive regulator of senescence and negative regulator of osteogenic differentiation of BMSCs [55].

Ubiquitin-conjugating enzyme E2E3 (*UB3E2E3*) in vitro knock-down in young BMSCs accelerated cellular senescence and inhibited osteogenic differentiation [58]. In addition, knockdown of *Bmi-1*, an important epigenetic regulator of stem cell self-renewal, leads to cellular senescence in young MSCs and overexpression to rejuvenation of old MSCs. Moreover, *Bmi-1* expression in MSCs was shown to be downregulated by elevated levels of the SASP component IL-1α in the aged bone marrow microenvironment [59]. Another mediator of BMSC senescence recently investigated is leucine-rich repeat containing 17 (LRRc17) [57]. *LRRc17* knockdown in BMSCs promoted osteogenic over adipogenic differentiation and activated mitophagy via inhibition of the mTOR/PI3K pathway. Autophagy preventing mitochondrial dysfunction was therefore suggested to be the mechanism leading to rejuvenation of senescent cells by LRRc17. An in vivo contribution of LRRc17 in promoting bone loss was suggested by an increased ovariectomy (OVX)-induced bone loss seen with old BMSCs transfected with shLRRc17 [57]. Moreover, results from Guo et al. [62] underline the role of mitophagy and BMSCs by suggesting a link between the accumulation of advanced glycation end products (AGEs) and BMSC senescence via Sirtuin-3 (SIRT3)-mediated mitophagy. Further, a contribution of this mechanism to bone loss is suggested by showing that in vivo *Sirt3* overexpression in SAMP6 mice reduces BMSC senescence and senile osteoporosis.

In a very recent study Li et al. [63] provide evidence for the accumulation of proinflammatory and senescent subtypes of immune cells in the bone marrow of aging rats and mice. These cells were shown to secrete granalcin, which in turn represses osteogenesis and promotes adipogenesis [63], thereby underlining the crucial interplay between the immune system and senescence in promoting age-related phenotypes. In another study Guo et al. [62] support a link between the accumulation of advanced glycation end products (AGEs) and BMSCs senescence by suggesting Sirt3-mediated mitophagy as regulatory mechanism of this process. Finally, two recent studies provide evidence for the important role of extracellular vesicles in the crosstalk of senescent cells of the bone compartment and non-bone cells [53,64]. Fulzele et al. have shown that EVs from mouse myoblasts and primary human myotubes with elevated levels of miR-34a induced senescence in primary mouse BMSCs. Moreover, an increased expression of the senescence-associated miR-34a was observed in skeletal muscle and serum extracellular vesicles of old versus young C57BL6 mice [53]. In another study by Lu et al., it was shown that exosome-mediated miR-139-5p derived from senescent osteoblasts regulates endothelial cell function [64]. This observation serves a better understanding of the widely accepted interplay of angiogenesis and osteogenesis in the pathology of osteoporosis.

Taken together, a major focus in recent research has been on the role of senescence in BMSC proliferation and differentiation, and major progress has been made in elucidating potential regulators of senescence-mediated bone loss in age-related osteoporosis. This knowledge provides an important foundation for an in-depth understanding of the application of already existing senescence-based therapeutic options in the treatment of osteoporosis. Furthermore, by closing the gaps, in future, novel therapeutic options with a more specific and individualized approaches may arise.

## 4. Treatment Options to Target Osteoporosis and Senescence

### 4.1. Osteoporosis-Specific Medication

Several osteoporosis-specific drugs are available. A meta-analysis of randomized controlled trials or post hoc analyses revealed a reduced incidence of vertebral fractures (RR = 0.43), non-vertebral fractures (RR = 0.84), and hip fractures (RR = 0.75) in the age group 75+ taking antiresorptives [65]. The limited data of patients 85 years or older suggest a continued efficacy of antiresorptives as well as teriparatide with an adverse reaction profile as in younger subjects [66]. Concerning the only drug available with a dual mode of action, romosozumab, up to now no post hoc analyses exist, but in the ARCH (Active Controlled Fracture Study of Postmenopausal Women with Osteoporosis at High Risk) study, half of the patients were 75 years or older [67]. Since patients included in the pivot trials of the osteoporosis-specific drugs supplemented calcium and vitamin D, co-administration of these two drugs is generally recommended. Vitamin D deficiency, as well as hypocalcemia, has to be resolved before starting a bone-specific medication. During bisphosphonate therapy, according to Reid [68], calcium supplementation may not be necessary, but denosumab and anabolic drugs should be prescribed.

All the above-mentioned drugs are approved for the treatment of osteoporosis. However, besides their action on bone metabolism, they may also have off-target effects. Fifteen years ago, a reduction in mortality was found in hip fracture patients receiving zoledronate (HR 0.72) [69]. A recent review described several potential positive non-skeletal effects of bisphosphonates besides overall mortality: a reduction in cardiovascular events and mortality as well as cancer incidence and mortality [70]. The center and co-authors suggested that the mechanisms leading to the extension of life expectancy are multifactorial including immunomodulatory effects [71].

Besides bone-specific medications, strategies targeting cellular senescence may be a treatment option for osteoporosis in the future. Elimination of senescent cells by a genetic approach in aged mice [35,42] led to the aim of therapeutically targeting the detrimental effects of senescence. That may be achieved by two different strategies: inhibition of the secretion of the senescence-associated secretory phenotype (SASP) or pharmacologic elimination of senescent cells.

### 4.2. Senostatics/Senomorphics

Senostatics modulating the proinflammatory senescence secretome are the so-called generalized senostatics. Ruxolitinib, an inhibitor of the Janus kinase pathway (JAKi) approved for the treatment of certain bone marrow disorders, suppresses multiple SASP components. The treatment of ruxolitinib led to amelioration of bone microarchitecture and increased bone strength in 22-month-old mice [42]. However, as SASP factors also have non-senescence related functions, negative off-target effects may accompany continuous inhibition of the SASP.

Inhibiting only the components of the SASP with deleterious effects may be superior. Thus, the components must be categorized according to their functions before precision senostatics can be taken into account for treatment. Up to now, precision senostatics have not been considered as therapeutic strategy for delaying age-related diseases.

### 4.3. Senolytics: Pre-Clinical Studies

In contrast to senostatics, senolytics—drugs that selectively promote apoptosis of senescent cells—may be given intermittently. Temporary suppression of pathways, which enable senescent cells to resist the pro-inflammatory and pro-apoptotic factors they secrete themselves, leads to the elimination of senescent cells. Several senolytics have been described by now [72]. Some of them may decrease age-associated bone loss (Table 1).

Dasatinib, an inhibitor of multiple tyrosine kinases, used for treating cancers [83] has been identified as a bone-modifying agent. The concomitant promotion of osteogenic differentiation as well as function and inhibition of osteoclast formation leads to a positive effect on bone in vivo shown by increased trabecular microarchitecture [73].

The flavonoid quercetin, which is present in foods (for instance, fruit, vegetables, tea, and wine), triggers apoptosis via the BCL-2 pathway. Quercetin showed to significantly reduce senescent rat BMSCs in vitro and to increase the osteogenic potential while decreasing the adipogenic potential [74]. According to two recent review articles, quercetin and its derivates ameliorate BMD and microarchitecture leading to an increase in maximum load [84,85]. These positive effects are reached by several different pathways. An interesting approach is the use of quercetin to improve osseointegration of implants under osteoporotic conditions; according to a rat model, it works [75].

Since dasatinib and quercetin target different molecular mechanisms in senescent cells, combining the two of them increases efficiency [86]. A calvarial defect model recently showed that this senolytic cocktail enhances the osteogenic potential of aged BMSCs [76]. An animal study detected the alleviation of radiation-induced bone loss by dasatinib and quercetin [51].

Navitoclax (ABT-263), a specific inhibitor of the anti-apoptotic proteins BLC-2 and BCL-xL, which are upregulated in senescent cells, proved to have senolytic effects in vitro and in vivo. It reduced the viability of some radiation-entailed senescent cell types [78], and it decreased the amount of aged or by total-body irradiation depleted senescent hematopoietic stem cells in mice [36]. Novitoclax-induced amelioration of apoptosis in BMSCs was underlined by the reduction in SASP in aged mice [79]. In human BMSCs, the senolytic effect was moderate [80].

Fisetin, a naturally occurring flavonoid with less toxicity than navitoclax [87] also proved to protect bone. Repression of osteoclasts [81] and promotion of osteoblast differentiation [82] seem to be the pathways responsible for the positive effects on BTMs, BMD, and bone microarchitecture.

### 4.4. Senolytics: Clinical Studies

Because of the positive results of senolytic agents in pre-clinical studies, translation into clinical interventions started. An open-label clinical study evaluating the combination of dasatinib and quercetin, which are known to influence bone metabolism in vitro as well as in mice, is ongoing (https://clinicaltrials.gov/ct2/show/NCT02848131; accessed on 24 February 2022). Preliminary data are already published: Oral intake of 100 mg dasatinib and 1000 mg quercetin for three days reduced blood SASP components and decreased the number of senescent cells in patients with diabetic kidney disease [88].

Another open-label phase 2 trial investigating the effect of senolytics on skeletal health is registered (https://clinicaltrials.gov/ct2/show/record/NCT04313634; accessed on 24 February 2022). One-hundred and twenty elderly women will be randomized to one of two experimental groups receiving either 100 mg dasatinib for two days and 1000 mg quercetin for three consecutive days starting every 28 days (five total dosing periods) or 20 mg/kg fisetin for three consecutive days on an intermittent schedule starting every 28 days (five total dosing periods), or a control group without any intervention. Primary outcome measures of this study are percent changes in serum levels of the bone resorption marker C-terminal telopeptide of type I collagen (CTX) and the bone formation marker amino-terminal propeptide of type I collagen (P1NP) within 20 weeks. Results for this study are not yet available.

### 4.5. Possible Adverse Effects of Senolytic Agents

Senescent cells have different (physiologic) functions including tumor suppression and beneficial effects on tissue repair, for instance, wound healing [89]. Thus, suppressing SASP as well as promoting apoptosis of senescent cells may have deleterious side effects. Unfortunately, navitoclax is toxic for platelets inducing transient thrombocytopenia and thrombocytopathy [90]. Galacto-conjugation of navitoclax seems to be an effective strategy increasing senolytic specificity and, thus, reducing platelet toxicity [91]. The clearance of senescent cells by dasatinib and quercetin given after inducing femoral fractures eliminated senescent cells and decreased SASP markers in the fracture callus, which are physiologically increased during fracture healing, but luckily did not impair, even accelerated fracture healing [92]. Dasatinib plus quercetin augmented bone regeneration of defects in rat calvaria after lipopolysaccharide sustained-release gelatin sponge (LS-G) implantation, which is supposed to increase stress-induced premature senescence (SIPS) cells [93].

Of course, we also have to be aware that the dosing regimen is important and not fully investigated. High concentrations of quercetin inhibited osteoblast differentiation in vitro and, thus, may induce an adverse effect on bone metabolism [77]. A recent study treating aged mice continuously with novitoclax (50 mg/kg/day) for two weeks, rather than intermittently, caused trabecular bone loss [94].

Exercise may be a non-pharmacologic senolytic without such adverse effects. It increases osteoblast and osteocyte function while reducing osteoclast activity; supposed modes of action discussed are SASP suppression as well as improved ROS (reactive oxygen species) defense [95].

## 5. Conclusions

Osteoporosis is a frequent age-related disease that results from a dysregulation of the activities of osteoclasts and osteoblasts. One of the hallmarks of aging is the accumulation of senescent cells; as in almost all tissues, also the cells of bone acquire a “senescence-associated secretory phenotype” (SASP). Since components of the SASP are involved in the pathophysiology of age-related osteoporosis, cellular senescence is a promising treatment target. In preclinical studies senolytic drugs showed favorable bone effects; translation into clinical interventions currently is under investigation.

## Figures and Tables

**Table 1:** Selected pre-clinical and clinical studies on bone effects of senolytics.

Agent	Setting		Intervention	Main Effects	Reference
**Pre-clincial**					
Dasatinib	In vitro	Human BMSCs	2–5 nM, 7 or 21 d	Tyrosine kinases (PDGFR-ß, c-SRC, c-Kit) ↓, canonical Wnt signaling pathway ↑	Garcia-Gomez et al. [73]
Dasatinib	In vitro	Human PBMCs	2–2.5 nM, 7 or 14 d	Oc differentiation↓ (c-Fos ↓, NFATc1 ↓), Oc function ↓ (cathepsin K ↓, αVß3 integrin ↓, CCR1 ↓)	Garcia-Gomez et al. [73]
Dasatinib	In vivo	Young mice	2.5 or 10 mg/kg p.o., 3 or 7 we	Serum levels of ALP ↑, Oc ↑, and TRAP5b ≈, osteoblast-like cells ↑, trabecular structure ↑	Garcia-Gomez et al. [73]
Quercetin	In vitro	Rat BMSCs	33.8 µg/mL, 24 h	senescent BMSCs ↓, BMSCs proliferation ↑, osteogenic potential ↑ (osterix ↑, RUNX2 ↑)	Zhang et al. [74]
Quercetin	In vivo	Rat model	Quercetin release system on titanium implants	Osseointegration ↑	Wang et al. [75]
Dasatinib + quercetin	In vivo	Aged C57BL/6 J mice	Dasatinib 5 mg/kg + quercetin 50 mg/kg p.o. per month, 4 months	Senescent osteocytes ↓, osteoclasts ↓, osteoblasts ≈, trabecular microarchitecture at spine and femur ↑, cortical microarchitecture at femur ↑	Farr et al. [42]
Dasatinib + quercetin	In vivo	C57BL/6 J mice	Dasatinib 5 mg/kg + quercetin 50 mg/kg p.o. 0 and 14 d after FRT of femur; bones harvested 42 d post FRT	P1NP ↑, CTX ≈, osteoblasts ↑, OCN ≈, RUNX2 ≈, BV/TV ↑, connectivity density ↑, BFR/BS ↑	Chandra et al. [51]
Dasatinib + quercetin	In vitro	BMSCs of young and old mice	Dasatinib 0.2 µM + quercetin 20 µM, 24 h	SABG + BMSCs ↓, BMSC proliferation ↑, OCN ↑, bone sialoprotein ↑	Zhou et al. [76]
Dasatinib + quercetin	In vivo	Immunodeficient mice	Old BMSCs: dasatinib 0.2 µM + quercetin 20 µM, 24 h => implanted into calvarial defect	TRAP ↑, ALP ↑, osteogenic capacity of aged BMSCs ↑	Zhou et al. [76]
Dasatinib + quercetin	In vitro	Mouse MSCs	Induction of senescence => Dasatinib 200 nM + quercetin 50 µM, 24 h	Senescence in MSCs ↓	Saul et al. [77]
Dasatinib + quercetin	In vivo	Mice	Dasatinib 5 mg/kg + quercetin 50 mg/kg p.o., 5 w	MSCs ↑, senescent MSCs ↓	Saul et al. [77]
Dasatinib + quercetin	In vivo	Mice	Dasatinib 5 mg/kg + quercetin 50 mg/kg once => induction of fracture p.o. => Dasatinib 5 mg/kg + quercetin 50 mg/kg p.o., 5 w	SASP in callus ↓, fracture healing time ↓, maximal torque after 2 w ↑	Saul et al. [77]
Navitoclax	In vitro	Radiation induced senesent HUVECs	100 nM to 1 uM up to 3 d	Apoptosis ↑; no effect on non-senesent cells	Zhu et al. [78]
Navitoclax	In vivo	Young sublethally irradiated and aged p16-3MR transgenic mice and C57/BL6 mice	50 mg/kg/d p.o., 2 cycles of 7 d, 2 w break in between	Number of HSCs and HPCs ≈, rejuvenates aged HSCs (for instance persistent DNA damage ↓); SASP ↓	Chang et al. [36]
Navitoclax	In vitro	Osteoprogenitor cells of old Osx1-Cre;TdRFP mice	5 µM, 5 d	Apoptosis of BMSCs ↑ SASP ↓, RANK L ↓	Kim et al. [79]
Navitoclax	In vitro	Human BMSCs	10 µM, 3 d	Senescent human BMSCs ↓	Grezella et al. [80]
Fisetin	In vitro	BMSCs of 3–5 week-old C57/BL6 mice, osteoclast precursors Raw264.7 cells	1–10 µM, 7/4 d	TRAP ↓, CTR ↓, MMP9 ↓, cathepsin K ↓, NF-kB pathway ↓, p38 MAPK/JNK ↓, c-FOS/NFATc1 ↓, MKP-1 ↑ => osteoclastogenesis ↓	Léotoing et al. [81]
Fisetin	In vivo	Young C57/BL6 mice	5–50 mg/kg p.o., 1 w => OVX => 5–25 mg/kg p.o., 4 w	BMD ↑, BV/TV ↑, TbN ↑, TbTh ↑	Léotoing et al. [81]
Fisetin	In vivo	Young C57/BL6 mice	LPS s.c. 1/w for 3 w and fisetin 5–50 mg/kg p.o.	BMD ↑, BV/TV ↑, TbN ↑	Leotoing et al. [81]
Fisetin	In vitro	MC3T3-E1 mouse preosteoblasts	0–800 nM, 14 d	ALP ↑, RUNX2 ↑, Col1α1 ↑, OSX ↑, OCN ↑, BMP4 ↑ => osteoblastogenesis ↑	Molagoda et al. [82]
Fisetin	In vitro	MC3T3-E1 mouse preosteoblasts	0–800 nM, 12 d after 2 d of 20 µM prednisolone	Osteoblast-specific gene expression restored, anti-osteoblastic genes (NFATc1, ACP, DC-STAMP, cathepsin K) downregulated	Molagoda et al. [82]
Fisetin	In vivo	Zebrafish larvae 3 dpf	50, 100, and 200 µM until 9 dpf	RUNX2a ↑, RUNX2b ↑, Col1α1 ↑, OSX ↑, OCN ↑, BMP4 ↑ => number of vertebrae ↑	Molagoda et al. [82]
**Clinical**					
Dasatinib + quercetin, fisetin	In vivo	120 females 70+	Dasatinib+quercetin vs. fisetin p.o, intermittently, 20 w.	Ongoing (outcome measures: BTMs)	NCT04313634

ACP: acid phosphatase; ALP: alkaline phosphatase; BFR/BS: bone formation rate per bone surface; BMP4: bone morphogenic protein 4; BMSCs: bone marrow mesenchymal stem cells; BTMs: bone turnover markers; BV/TV: bone volume per tissue volume; CCR1: C-C chemokine receptor 1; c-FOS: key transcription factors; Col1α1: collagen type 1 alpha 1; CTR: calcitonin receptor; d: days; DC-STAMP: dendritic cell-specific transmembrane protein; dpf: days post-fertilization; FRT: focal radiation treatment; HPCs: hematopoietic progenitor cells; HSCs: hematopoietic stem cells; HUVECs: human umbilical vein epithelial cells; JNK: c-jun-N-terminal kinase; MKP-1: MAPK phosphatase 1; MMP9: matrix metalloproteinase 9; MSCs: mesenchymal stromal cells; NFATc1: nuclear factor of activated T cells 1; NF-kB: nuclear factor kB; NTX: N-telopeptide; Oc: osteoclast; OCN: osteocalcin; OSX: osterix; OVX: ovariectomy; p38 MAPK: p38 mitogen-activated protein kinase; PBMCs: peripheral blood mononuclear cells; RANKL: receptor activator nuclear factor kB; RUNX2: runt-related transcription factor 2; SABG: senescence-associated beta-galactosidase; SASP: senescence-associated secretory phenotype; TRAP: tartrate resistant acid phosphatase; w: weeks; ↓: decreased; ↑: increased; => leads to.
